# A Mediterranean lifestyle is associated with favourable cardiometabolic markers in people with non-dialysis dependent chronic kidney disease

**DOI:** 10.1017/jns.2021.33

**Published:** 2021-06-04

**Authors:** Katelyn Bowden, Nicholas A Gray, Elizabeth Swanepoel, Hattie H Wright

**Affiliations:** 1 School of Health and Behavioural Sciences, University of the Sunshine Coast, Maroochydore, Queensland, Australia; 2 Department of Nephrology, Sunshine Coast Hospital and Health Service, Birtinya, Queensland, Australia; 3 Sunshine Coast Health Institute, Birtinya, Queensland, Australia

**Keywords:** Dietary components, Dietary habits, Kidney disease, Lifestyle behaviour, Mediterranean diet

## Abstract

Adherence to a Mediterranean lifestyle may be a useful primary and secondary prevention strategy for chronic kidney disease (CKD). This cross-sectional study aimed to explore adherence to a Mediterranean lifestyle and its association with cardiometabolic markers and kidney function in 99 people aged 73⋅2 ± 10⋅5 years with non-dialysis dependant CKD (stages 3–5) at a single Australian centre. Adherence was assessed using an *a priori* index, the Mediterranean Lifestyle (MEDLIFE) index. Cardiometabolic markers (total cholesterol, LDL-cholesterol, HbA1c and random blood glucose) and kidney function (estimated GFR) were sourced from medical records and blood pressure measured upon recruitment. Overall, adherence to a Mediterranean lifestyle was moderate to low with an average MEDLIFE index score of 11⋅33 ± 3⋅31. Adherence to a Mediterranean lifestyle was associated with employment (*r* 0⋅30, *P* = 0⋅004). Mediterranean dietary habits were associated with cardiometabolic markers, such as *limiting sugar in beverages* was associated with lower diastolic blood pressure (*r* 0⋅32, *P* = 0⋅002), *eating in moderation* with favourable random blood glucose (*r* 0⋅21, *P* = 0⋅043), *having more than two snack foods per week* with HbA1c (*r* 0⋅29, *P* = 0⋅037) and LDL-cholesterol (*r* 0⋅41, *P* = 0⋅002). Interestingly, *eating in company* was associated with a lower frequency of depression (*χ*^2^ 5⋅975, *P* = 0⋅015). To conclude, Mediterranean dietary habits were favourably associated with cardiometabolic markers and management of some comorbidities in this group of people with non-dialysis dependent CKD.

## Introduction

Chronic kidney disease (CKD) affects 9⋅1 % of the population worldwide, placing substantial burden on healthcare systems^(1,2)^. The development and rapid progression of kidney dysfunction is associated with sub-optimal dietary intake^(3)^. Not only is low diet quality detrimental to CKD, it can also result in poor management of underlying comorbidities like cardiovascular disease, diabetes mellitus and hypertension which can further progress CKD^(3,4)^. For these reasons, there is potential for healthful dietary patterns, like the Mediterranean diet, to aid in the prevention and management of CKD^(5)^.

Emerging evidence links a Mediterranean diet with improved cardiometabolic health, glycaemic control and reduced incidence of CKD^(5–8)^. The Mediterranean diet is based on high intake of fruits, vegetables, whole-grain bread and cereals, olive oil, legumes, nuts and seeds; moderate intake of dairy products, egg, seafood, poultry and wine; low intake of red meat and discretionary foods^(9)^. In a multi-centre trial, a Mediterranean diet supplemented with extra-virgin olive oil or nuts was associated with a 31 % lower incidence of major cardiovascular events in people at high cardiovascular risk^(8)^. Moreover, the Mediterranean diet has been associated with lower blood pressure, low-density lipoprotein and higher estimated glomerular filtration rate (eGFR) in people with CKD^(10)^. Likewise, adherence to a Mediterranean diet was associated with reduced levels of HbA1c, plasma glucose and insulin in adults with and without type 2 diabetes mellitus^(11,12)^. Lastly, a recent prospective cohort study among CKD patients found that those with highest adherence to a Mediterranean diet had a lower adjusted risk for CKD progression^(13)^. Taken together, a Mediterranean diet seems to be a prudent diet for people at risk of or with CKD, due to its impact on comorbidities, which may contribute to the development or progression of CKD.

The protective effects of a Mediterranean diet may not only be due to the intake of nutrients and foods but also include non-nutrient factors such as psychological, social and physical factors that represent a Mediterranean lifestyle^(14,15)^. These factors have been shown to modulate comorbidities associated with CKD and to a lesser extent, CKD and its progression^(16,17)^. Physical activity is well documented to decrease body weight, blood pressure, low-density lipoprotein and total cholesterol levels^(15,18–21)^. Moreover, sedentary behaviour and reduced physical activity are associated with an increased risk of CKD, as well as increased mortality and decline in renal function in CKD populations^(15,19)^. Socialisation has been identified to be inversely associated with the presence of hypertension^(17)^ and depression^(22)^. Lastly, conviviality has been associated with healthy weight, improved eating habits and dietary choices^(23)^. Taken together, it appears the benefits of a Mediterranean lifestyle are not exclusive to dietary intake; thus, further consideration to its role in the health outcomes and progression of kidney disease in people with non-dialysis dependent CKD is warranted.

The aim of the present study was to investigate the adherence to a Mediterranean lifestyle and its association with cardiometabolic markers and kidney function in people with non-dialysis dependent CKD from a non-Mediterranean country.

## Methods

### Study design and setting

This cross-sectional observational study was conducted at the Nephrology department of the Sunshine Coast Hospital and Health Service, Queensland, Australia. People with non-dialysis dependent CKD stages 3 to 5 (eGFR ≤60 ml/min/1⋅73 m^2^) were recruited via convenience sampling. The CKD stage was determined according to the National Kidney Foundation clinical practice guidelines^(24)^. The primary outcome was adherence to a Mediterranean lifestyle and secondary outcomes were kidney function and cardiometabolic health. Dependent variables were kidney function (eGFR) and cardiometabolic markers (total cholesterol, LDL-cholesterol, HbA1c, random blood glucose and blood pressure). Independent variables included Mediterranean lifestyle adherence, socioeconomic status, the presence of comorbidities, age, weight and body mass index (BMI). Exclusion criteria included kidney transplant recipients, people receiving dialysis, non-English-speaking individuals, cognitive impairment, pregnancy and current enrolment in another research project. Nil reimbursement or incentives were provided. Participants provided written informed consent prior to inclusion to the study. Ethical approval was obtained from the Prince Charles Hospital Human Research Ethics Committee (HREC/17/QPCH/370) and the University of the Sunshine Coast Human Research Ethics committee (S181179).

### Measurements

Height, weight and brachial blood pressure were measured by trained clinical nurses using standard protocols^(25,26)^ during a routine visit at the Nephrology department. BMI was calculated from height and weight measurements and categorised according to the World Health Organisation Criteria (Healthy: BMI ≥ 18⋅5 to <25 kg/m^2^; Overweight: BMI ≥ 25 to <30 kg/m^2^; Obese: BMI ≥ 30 kg/m^2^)^(27)^. The most recent biochemical parameters (taken within the last 6-months), the number of diagnosed comorbidities (including diabetes, cardiovascular disease, hypertension and depression) and medication use were recorded from medical charts.

### Questionnaire

Socio-demographic information, source of nutrition information and Mediterranean lifestyle habits were assessed using a self-administered paper-based survey.

#### Socio-demographic information

Socio-demographic data included age, sex, country of birth, employment, educational attainment, marital status, previous dietetic input and postcode. Employment status and level of education were surrogates for socioeconomic status.

#### Mediterranean lifestyle habits

The Mediterranean lifestyle (MEDLIFE) index^(28)^ was used to assess adherence to a Mediterranean lifestyle, including diet, physical activity patterns and social interaction. The MEDLIFE index has been validated in a middle-aged Spanish working-class cohort and showed significant association with the Alternate Mediterranean Diet Index and the Mediterranean Diet Adherence Screener^(28)^. The index consists of 28-items which are divided into three blocks, namely (1) food consumption (fifteen items), (2) traditional Mediterranean dietary habits (seven items) and (3) physical activity, rest, social habits and conviviality (six items). In block one, participants self-reported the average number of serves of specific foods and beverages using visual guides on portion sizes which was provided by the research team. Block two and three indicated adherence to Mediterranean dietary and lifestyle habits, respectively. Each item was scored one point if the guideline was met or zero if the guideline was not met, resulting in a total score out of 28. The higher the score, the greater adherence to a Mediterranean lifestyle is.

#### Sample size estimation

A total sample size of 102 was calculated using G-power^(29)^ with a medium effect size (*δ* 0⋅50), 80 % power and significance set at *P* < 0⋅05 to identify differences between two independent groups. A total of 111 participants were recruited between July and September 2020, of which 12 participants did not return their surveys, resulting in a final sample size of 99 (89 % response rate).

### Statistical analysis

Data analysis was performed using IBM SPSS statistical software package version 24 (SPSS, Inc., Chicago, IL, USA) and significance set at *P* < 0⋅05. Descriptive statistics are presented as mean  and standard deviation (SD) for parametric variables or median and interquartile range (IQR) for non-parametric variables. Categorical data are presented as frequencies and percentage of the total group. Data were stratified into dichotomous groups according to the median MEDLIFE index score of the cohort (cut-off: ≤11 *v.* >11). Additionally, participants were stratified based on kidney function, early (stages 3a and 3b) or advanced CKD (stages 4 and 5) to explore differences in socio-demographic and cardiometabolic markers. Differences between groups were determined with independent *T*-tests for parametric variables, Mann–Whitney *U* tests for non-parametric variables and *χ*^2^ test for categorical variables. Associations between independent and dependent variables were explored using *χ*^2^ test or Pearson correlations, adjusting for age, gender and weight. Adjusted *R*-values are reported unless otherwise indicated.

## Results

### Participant characteristics and cardiometabolic markers

Participant demographics and comorbidities are summarised in Table 1 for the total group, as well as between groups based on the MEDLIFE index score and kidney function. Participants were mostly male, older than 70 years of age, unemployed and the majority obtained either a secondary or tertiary education. Overall, 32⋅6 % were classified as overweight and 50⋅0 % were obese. Most participants presented with two or more comorbidities, with only 7⋅3 % of participants presenting with no comorbidity and 25⋅0 % presenting with one comorbidity. Significant differences were found between systolic blood pressure and a diagnosis of hypertension and/or cancer between those with early CKD compared with advanced CKD.

**Table 1.** Participant demographics and comorbidities (*n* 99)

	Total group (*n* 99)		MEDLIFE index score ≤ Median (*n* 50)		MEDLIFE index score > Median (*n* 47)		CKD stage 3 (*n* 47)		CKD stages 4 to 5 (*n* 52)	
Characteristics	Mean/Median	sd/IQR	Mean/Median	sd/IQR	Mean/Median	sd/IQR	Mean/Median	sd/IQR	Mean/Median	sd/IQR
Age (years)	73⋅2	10⋅5	72⋅1	10⋅4	74⋅1	10⋅7	72⋅7	10⋅2	74⋅0	10⋅8
Body weight (kg)	88⋅2	18⋅8	88⋅7	16⋅8	88⋅0	21⋅1	88⋅3	20⋅6	88⋅1	17⋅4
Body mass index^a^ (kg/m^2^)	30⋅0	26⋅2, 34⋅4	30⋅7	26⋅1, 35⋅0	28⋅3	26⋅2, 33⋅1	29⋅0	26⋅1, 34⋅7	30⋅6	26⋅2, 34⋅3
Systolic blood pressure^b^ (mmHg)	140	20⋅0	141	19⋅2	140	20⋅7	133*	19⋅6	146	18
Diastolic blood pressure^b^ (mmHg)	76	8⋅5	77	7⋅2	75	9⋅5	75	9⋅3	6⋅4	7⋅7
eGFR^b^ (ml/min/1⋅73 m^2^)	30	13⋅1	29	13⋅0	31	3⋅7	41⋅6*	8⋅9	20⋅1	6⋅1

	*N*	%	*N*	%	*N*	%	*N*	%	*N*	%
Married	50	50	26	52	24	51	20	43	30	58
Male	64	64	35	70	28	60	27	59	36	69
Employed^c^	13	13	4	8	9	19	5	11	7	13
Education										
No formal	16	16	7	14	9	19	10	22	6	12
Secondary	44	44	21	42	22	47	20	43	24	46
Tertiary or vocational	39	40	22	44	16	34	16	35	22	42
Presence of comorbidities^d^										
Hypertension	64	65	31	62	32	68	24	52^#^	40	77
CVD	49	50	28	56	20	43	21	46	28	54
Diabetes	51	52	24	48	27	57	20	43	31	60
Cancer	20	20	8	16	11	23	5	11^$^	15	29
Depression	19	19	10	20	8	17	12	26	7	13


*Note*. Data presented as mean and standard deviation (SD), median and interquartile range (IQR, 25th; 75th percentile), or frequency and percentage of the total group.

MEDLIFE, Mediterranean lifestyle; CKD, chronic kidney disease; eGFR, estimated glomerular filtration rate; CVD, cardiovascular disease.

a Data missing for thirteen participants.

b Data missing for one participant.

c Includes full-time, part-time and casual employment.

d Participants presented with multiple comorbidities; values do not add up to 100.

* Significant difference between CKD stage 3 compared with stages 4 and 5, *P* < 0⋅01.

# *χ*^2^ 6⋅599, *P* = 0⋅01.

$ *χ*^2^ 4⋅856, *P* = 0⋅028.

Table 2 shows biochemistry, MEDLIFE index score and cardiometabolic markers for the group. No significant differences were found for cardiometabolic markers or kidney function between groups according to the median MEDLIFE index score. Significant differences were found in creatinine, potassium, phosphate and triacylglycerols between those with early CKD compared with advanced CKD.

**Table 2.** Participant biochemistry, cardiometabolic markers and Mediterranean lifestyle index (*n* 99)

	Total group (*n* 99)		MEDLIFE index score ≤ Median (*n* 50)		MEDLIFE index score > Median (*n* 47)		CKD stage 3 (*n* 47)		CKD stages 4 to 5 (*n* 52)	
Measurements and characteristics	Mean	sd	Mean	sd	Mean	sd	Mean	sd	Mean	sd
MEDLIFE Index score	11⋅3	3⋅3	8⋅8	1⋅9	14⋅0	2⋅1	11⋅8	2⋅9	10⋅8	3⋅5
Creatinine^a^ (umol/l)	203	89⋅3	210	88⋅3	198	91⋅7	134*	21⋅3	265	80⋅7
Albumin^b^ (g/l)	40	3⋅7	40	3⋅7	40	3⋅7	40	3⋅7	40	3⋅7
Potassium^a^ (mmol/l)	4⋅5	0⋅5	4⋅6	0⋅5	4⋅5	0⋅5	4⋅4*	0⋅4	4⋅7	0⋅5
Phosphate^a^ (mmol/l)	1⋅2	0⋅2	1⋅2	0⋅2	1⋅2	0⋅3	1⋅1*	0⋅2	1⋅3	0⋅2
Random blood glucose^b^ (mmol/l)	7⋅1	3⋅0	7⋅3	3⋅2	6⋅9	2⋅8	7⋅0	2⋅8	7⋅1	3⋅1
HbA1c^c^ (%)	7⋅5	1⋅9	7⋅5	2⋅2	7⋅5	1⋅5	7⋅3	1⋅8	7⋅6	2⋅0
Total cholesterol^c^ (mmol/l)	4⋅4	1⋅2	4⋅2	1⋅1	4⋅5	1⋅3	4⋅3	1⋅2	4⋅5	1⋅3
HDL-cholesterol^d^ (mmol/l)	1⋅1	0⋅3	1⋅1	0⋅3	1⋅1	0⋅3	1⋅2	0⋅3	1⋅0	0⋅3
LDL-cholesterol^d^ (mmol/l)	2⋅1	0⋅9	2⋅1	0⋅9	2⋅1	0⋅8	2⋅2	0⋅9	2⋅1	0⋅9
Triacylglycerols^e^ (mmol/l)	1⋅9	0⋅9	1⋅9	0⋅8	1⋅9	1⋅0	1⋅7**	0⋅7	2⋅1	1⋅1

*Note*. Data presented as mean and standard deviation (SD).

MEDLIFE, Mediterranean lifestyle; CKD, chronic kidney disease; HbA1c, glycated haemoglobin A1C; HDL, high-density lipoprotein; LDL, low-density lipoprotein.

a Data missing for one participant.

b Data missing for three participants.

c Data missing for seven participants, nil readings within 6 months.

d Data missing for forty participants, nil readings within 6 months.

e Data missing for thirteen participants, nil readings within 6 months.

* Significant difference between CKD stage 3 compared with stages 4 and 5, *P* < 0⋅01.

** Significant difference between CKD stage 3 compared with stages 4 and 5, *P* < 0⋅05.

The most common source of nutrition information was a dietitian (30⋅3 %), doctor (29⋅3 %), friends or family (29⋅3 %) and the internet or online resources (21⋅2 %).

### Mediterranean lifestyle habits

Two participants completed less than 70 % of the MEDLIFE index and were omitted from data analysis. The average MEDLIFE index score for the total group was 11⋅3 ± 3⋅3 (*n* 97). Table 3 represents the average scores for each block, as well as individual items of the MEDLIFE index.

**Table 3.** Adherence to a Mediterranean lifestyle, the MEDLIFE index raw scores (*n* 97)

		Criteria for 1 point^a^	Average serves^b^	Participants who met criteria		
			Mean	sd	*N*	%
*Block 1: Mediterranean food consumption*						
1. Sweets	Candy (1s = 1 unit or 50 g), chocolates (1s = 30 g), biscuits (1s = 4–6 units)	≤2 s/week	6⋅6	6⋅2	26	26⋅3
2. Red meat	Beef, pork, kangaroo, lamb (1s = 90–100 g)	<2 s/week	4⋅0	2⋅7	41	41⋅4
3. Processed meat	Ham/salami (1s = 1 slice or 30 g), sausage, bacon (1s = 50 g/2 rasher), hamburger (1s = 1 patty), liver (1s = 100–150 g), pate (1s = 25 g)	≤1 s/week	2⋅9	3⋅1	20	20⋅2
4. Eggs	Eggs (1 egg)	2–4 s/week	3⋅7	3⋅1	39	39⋅4
5. Legumes	Lentils, beans, peas, chickpeas (1s = 1 cup or 150 g)	≥2 s/week	0⋅9	1⋅4	22	22⋅2
6. White meat	Poultry, rabbit (1s = 100–150 g)	2 s/week	2⋅3	1⋅4	21	21⋅2
7. Fish/seafood	White/oily fish (1s = 100–150 g), canned fish (1s = 1 can or 50 g), seafood (1s = 200 g)	≥2 s/week	1⋅4	1⋅2	35	35⋅4
8. Potatoes	Roast/boiled potatoes, French fries/hot chips (1s = 150–200 g)	≤3 s/week	4⋅4	2⋅9	40	40⋅4
9. Low-fat dairy	Skimmed dairy milk (1s = 250 ml milk, two small yoghurts or 200 g tub, 1 portion cheese)	2 s/d	1⋅0	1⋅2	12	12⋅1
10. Nuts and olives	Walnuts, almonds, hazelnuts (1s = 1 handful or 30 g), olives (1s = 10 units)	1–2 s/d	0⋅3	0⋅8	24	24⋅2
11. Herbs, spices, garnish	Onion, garlic, herbs (parsley, oregano)	≥1 s/d	0⋅6	0⋅8	54	54⋅5
12. Fruit	All fruit and fresh fruit-based juices (1s = 150–200 g or 125 ml juice)	3–6 s/d	1⋅6	1⋅3	14	14⋅1
13. Vegetables	All vegetables except potatoes (1s = 150–200 g)	≥2 s/d	2⋅1	1⋅3	62	62⋅6
14. Olive oil	Olive oil (1s = 1 tbsp)	≥3 s/d	0⋅8	1⋅7	4	4⋅0
15. Cereals	White and whole-grain bread (1s = 1 slice or 40 g), cereals (1s = 1/2 cup rice, pasta) or (1s = 1/2 cup or small bowl of porridge, breakfast cereals) and derivatives	3–6 s/d	2⋅8	1⋅7	49	49⋅5
*Block 2: Mediterranean dietary habits*						
16. Water or infusions	Water or infusions (1 serving = 1 glass)	>6 s/d	–	–	62	62⋅6
17. Wine at mealtimes	White/red wine (1s = 1 glass of wine)	Yes	–	–	8	8⋅1
18. Limit salt in meals		Yes	–	–	70	70⋅7
19. Preference for whole grains		Yes	–	–	50	50⋅5
20. Limit snacks	Potato chips, tortilla chips, popcorn (1s = 1 bag or 50 g)	<2 s/week	–	–	62	62⋅6
21. Limit nibbling between meals		Yes	–	–	73	73⋅7
22. Limit sugar in beverages		Yes	–	–	80	80⋅8
a. Consume seasonal/minimally processed food^c^		Yes	–	–	76	76⋅8
b. Consume small portions and in moderation^c^		Yes	–	–	71	71⋅7
*Block 3: Physical activity, rest, social habits and conviviality*						
23. Physical activity	Jogging, walking at a fast pace, dance/aerobics, gardening, >150 min/week or 30 min/d	Yes	–	–	46	46⋅5
24. Siesta/nap	During weekends	Yes	–	–	66	66⋅7
25. Hours of sleep	During weekdays	6–8 h/d	6⋅9	2⋅3	52	52⋅5
26. Watching television	During weekdays	≤1 h/d	5⋅0	6⋅8	10	10⋅1
27. Socialising with friends	During weekends	≥2 h/week	2⋅6	3⋅6	46	46⋅5
28. Collective sports	During week	≥2 h/week	0⋅2	1⋅0	3	3⋅0
c. Lunch time	During weekdays^c^	≥20 min/d	25⋅1	18⋅8	59	59⋅6
d. Eat in company	Family, friends, colleagues^c^	Yes	–	–	56	56⋅6

*Note*. S, serves.

a 0 points if criteria were not met.

b Data presented as mean and standard deviation (SD), or frequency and percentage of the total group.

c Questions denoted with a letter (a, b, c, d) are supplementary questions of the MEDLIFE index and are not included in the final index score.

Considering the recommended cut-offs for Mediterranean food consumption, on average, the number of serves for vegetables was met and most participants used herbs and spices to flavour food. In contrast, the number of serves for pastries, red meat, processed meat and potatoes were in excess, while the number of serves for legumes, low-fat dairy, olive oil, nuts, fruit, fish and seafood were not met. The majority of participants met the criteria for Mediterranean dietary habits with the exception of drinking wine daily at mealtimes. Over half of the participants met criteria for most Mediterranean lifestyle habits, excluding television viewing, participation in team sports or physical activity and socialising with friends.

### Correlations

#### Socio-demographics and Mediterranean lifestyle

The total MEDLIFE index score was associated with employment which remained significant after adjusting for age and gender (*r* 0⋅30, *P* = 0⋅004). An association between women and *preference to consume fresh, minimally processed and seasonal produce* (*χ*^2^ 6⋅525, *P* = 0⋅011), as well as *preference to eat in moderation and trying to choose small portions* (*χ*^2^ 10⋅371, *P* < 0⋅01) was found. There was an association between men and *eating in company* (*χ*^2^ 6⋅047, *P* = 0⋅014). Previous dietetic input was associated with *consumption of fresh, minimally processed and seasonal produce (χ*^2^ 3⋅670, *P* = 0⋅055).

#### Cardiometabolic markers, kidney function and Mediterranean lifestyle

Kidney function was negatively associated with systolic blood pressure which remained significant after adjusting for confounders (*r* −0⋅32, *P* = 0⋅002). Kidney function was associated with not receiving nutrition information from the internet or online sources (*r* 0⋅23, *P* = 0⋅026).

The total MEDLIFE index score was negatively associated with diastolic blood pressure but lost significance after adjusting for confounders (*r* −0⋅20, *P* = 0⋅058). Adherence *to limiting sugar in beverages* was associated with lower diastolic blood pressure before and after adjusting for confounders (*r* 0⋅32, *P* = 0⋅002).

Body weight was negatively associated with HDL-cholesterol (*r* −0⋅40, *P* = 0⋅002). Not adhering to *consumption of less than two servings of snack foods per week* was associated with LDL-cholesterol after adjusting for confounders (*r* 0⋅41, *P* = 0⋅002). An association was also found with total cholesterol, but significance was lost after adjusting for confounders (*r* 0⋅21, *P* = 0⋅054). Interestingly, *preference of minimally processed and seasonal produce* and *preference to eat in moderation and choosing small portions* was inversely associated with total cholesterol after adjusting for confounders (*r* −0⋅25, *P* = 0⋅019; *r* −0⋅22, *P* = 0⋅04, respectively).

Random plasma glucose was associated with non-adherence to *eating in moderation and trying to choose small portions* and remained significant after adjusting for confounders (*r* 0⋅21, *P* = 0⋅043). In addition, there was an association between participants presenting with diagnosed diabetes and non-adherence to *preference to eat in moderation and trying to choose small portions* (*χ*^2^ 4⋅174, *P* = 0⋅041). Not adhering to c*onsumption of less than two servings of snack foods per week* was associated with HbA1c and remained significant after adjusting for confounders (*r* 0⋅29, *P* = 0⋅037).

Interestingly, *eating in company* was associated with a lower frequency of diagnosed depression (*χ*^2^ 5⋅975, *P* = 0⋅015).

## Discussion

The present study aimed to investigate adherence to a Mediterranean lifestyle and its association with cardiometabolic markers and kidney function in people with non-dialysis dependent CKD. Key findings include moderate adherence to a Mediterranean lifestyle, employment was associated with higher adherence to a Mediterranean lifestyle, components of Mediterranean dietary habits were associated with various cardiometabolic markers and those that ate with others had a lower occurrence of depression. No associations between kidney function and a Mediterranean lifestyle were found.

Evidence points toward an association between adherence to a Mediterranean diet, renal function and delayed CKD progression^(5,30)^. In a systematic review of nine studies, increased adherence to a Mediterranean diet was significantly associated with a 10 % reduced risk of CKD in community-dwelling adults^(31)^. Additionally, a cross-sectional study (*n* 2813) found that greater adherence to a Mediterranean diet was associated with higher eGFR in people with CKD stage 3^(32)^. No significant associations were observed between a Mediterranean lifestyle adherence and CKD in the present study. This may be partly explained by the small sample size and the use of different tools to measure adherence to a Mediterranean diet and/or lifestyle. The present study assessed food intake, dietary habits, physical activity and social interaction which differs from the tools used in previous studies (e.g. Mediterranean Diet Score, Mediterranean Diet Adherence Screener, Mediterranean Diet Scale, Short Mediterranean Diet Questionnaire) that predominately focused on food and/or nutrients^(33)^. Lifestyle factors such as physical activity and sedentary behaviour may be useful in the prevention of CKD progression as well as comorbidities such as cardiovascular disease which are common among people with CKD^(15,18–20)^. Therefore, the present study used an *a priori* index tool that included some of these lifestyle factors. Despite the lack of associations between kidney function and a Mediterranean lifestyle, we did find associations between Mediterranean lifestyle components and cardiometabolic markers which may act to modulate CKD progression^(34)^. Nevertheless, the role of a Mediterranean lifestyle in the prevention of CKD and CKD progression requires further investigation.

Proposed strategies to attenuate CKD development and progression is to effectively manage risk factors of CKD, including underlying comorbidities like cardiovascular disease, hypertension and diabetes^(16,34,35)^. Our findings demonstrated that adherence to a Mediterranean lifestyle, especially Mediterranean dietary habits, was associated with favourable cardiometabolic health markers. These findings are supported by a meta-analysis of eleven studies^(36)^ which reported that in non-CKD populations those with the highest adherence to a Mediterranean diet had 24 % lower risk of incident cardiovascular disease and 28 % lower risk of incident coronary heart disease. In agreement, a recent meta-analysis of six trials showed that the Mediterranean diet had positive effects on cardiovascular parameters, including total cholesterol and blood pressure^(12)^. Likewise, a meta-analysis of nine randomised control trials demonstrated that a Mediterranean diet had beneficial effects on weight and cardiovascular risk factors (total cholesterol, triacylglycerols and blood pressure) in people with type 2 diabetes mellitus^(11)^. Furthermore, a parallel-group, intervention trial that assessed the efficacy of Mediterranean diets on primary cardiovascular prevention (PREDIMED study) among 7447 people, a Mediterranean diet supplemented with extra-virgin olive oil or nuts, was associated with a lower rate of major cardiovascular events (myocardial infarction, stroke or death from cardiovascular causes) compared with a reduced-fat diet^(8)^. Similarly, a cross-sectional study in Australia found that higher adherence to a Mediterranean diet was associated with lower total cholesterol and LDL-cholesterol levels, systolic blood pressure and a lower risk of dyslipidaemia^(20)^. Taken together, a Mediterranean diet is effective to improve cardiovascular health, potentially reducing CKD development and progression^(16)^. Notably, the role of dietary habits and other lifestyle factors, such as the physical lifestyle environment, was not assessed. A surprising result from the present study was the unexpected inverse association between total cholesterol and some Mediterranean dietary habits. This may be explained by the potential 6-month lag between the measurement of biochemical parameters and completion of the survey. Furthermore, perhaps individual dietary preferences did not necessarily translate into action.

Interestingly, an association between eating in company and lower presence of depression was identified in the present study. Eating in company represents social support and a sense of community; thus, conviviality of meals may positively affect dietary behaviour and, in turn, health status^(14)^. In fact, high adherence to a Mediterranean diet and socialisation was associated with a lower risk of developing depression in a prospective cohort study of Spanish adults^(22)^. The present study, therefore, supports the notion that non-nutritional aspects of a Mediterranean lifestyle, such as social and cultural factors, are fundamental to help procure the well-known benefits of a Mediterranean lifestyle.

In the present study, adherence to certain Mediterranean dietary habits was associated with improved glycaemia and a lower frequency of diagnosed type 2 diabetes mellitus. Our findings are in agreement with the body of evidence on the Mediterranean diet and glycaemia^(11,12)^. A meta-analysis of nine studies, investigating the effects of a Mediterranean diet on glycaemic control and cardiovascular risk factors, highlighted that adherence was associated with reduced levels of HbA1c, fasting glucose and insulin among patients with type 2 diabetes^(11)^. Similarly, a meta-analysis of six studies found that a Mediterranean diet had positive effects on plasma glucose in most healthy adults^(12)^. It is concluded that management of type 2 diabetes mellitus may improve with adherence to a Mediterranean diet, thereby slowing renal function decline.

Non-adherence to a Mediterranean lifestyle was observed in the present study. Adherence may be influenced by contextual factors, including the fact that participants were from a non-Mediterranean country, as well as factors relating to food access, food acceptance and socio-cultural norms^(18,37)^. Difficulty adhering to a Mediterranean diet within Australia, with the absence of an intensive intervention, was highlighted in a recent systematic review^(37)^. Potential avenues to improve adherence included the translation of key Mediterranean diet principles into the Australian Dietary Guidelines, thus acknowledging socio-cultural norms, sustainability, acceptability and palatability^(37)^. Nevertheless, further research is warranted into the transferability of a Mediterranean diet and lifestyle into recommendations for non-dialysis dependent CKD patients from non-Mediterranean countries while maintaining associated health benefits. Employment was also linked with higher adherence to a Mediterranean lifestyle in the present study. Evidence suggests improved health outcomes for older adults that maintain some form of employment status after the age of 65 years^(37,38)^. With this in mind, the role of employment in achieving and maintaining a healthier lifestyle in older adults with non-dialysis dependent CKD needs further investigation.

The limitations of the present study include a small sample size, a single centre study and the use of self-reported tools which can result in reporting errors or bias^(39–41)^. Furthermore, there was potential for up to a 6-month variance between measured biochemical parameters and data collection within the research study. Due to financial constraints, fasting blood samples could not be taken at the time of the study but were sourced from medical records. Retirement was not recorded, and it was, therefore, not possible to distinguish between unemployment due to retirement or due to inability to secure work. As the majority of participants (71⋅7 %) were 65-years and older, it is possible that many were retired; however, it cannot be assumed. In addition, the cross-sectional nature of the study design precludes conclusions pertaining to causality between a Mediterranean lifestyle, kidney function and cardiometabolic markers^(42)^.

## Conclusion

In summary, despite only moderate adherence to a Mediterranean lifestyle, components of a Mediterranean lifestyle were associated with improved health outcomes in this group of people with non-dialysis dependent CKD. The present study extends the body of knowledge by highlighting the potential role of Mediterranean lifestyle factors such as dietary habits, the physical environment and social interaction on cardiometabolic markers, kidney function and/or other health outcomes in people with non-dialysis dependent CKD. Further studies are warranted in larger samples investigating the role of both dietary and non-dietary Mediterranean lifestyle factors in the progression of CKD, as well as factors influencing adherence. In addition, validated tools measuring Mediterranean lifestyle quality, contextualised for people from non-Mediterranean countries, are needed.
